# Maternity Experiences and Perceptions of Emergency Medicine Physicians

**DOI:** 10.51894/001c.22009

**Published:** 2021-04-13

**Authors:** Lindsey McDonald, Zachary Illg, Agata Dow, Satheesh Gunaga

**Affiliations:** 1 Emergency Medicine, Henry Ford Wyandotte Hospital; Emergency Medicine, Fairview Ridges Hospital; 2 Emergency Medicine Henry Ford Wyandotte Hospital

**Keywords:** return to work, emergency medicine, female, maternity, breastfeeding

## Abstract

**INTRODUCTION:**

Postpartum employment has been recognized as a significant obstacle to breastfeeding continuation rates in the general population. Multiple additional factors can influence emergency medicine (EM) physician mothers’ ability to continue breastfeeding upon return to work. These include the unpredictable nature of emergency room volumes and acuity, absence of protected lactation time or facilities, and varying levels of support from colleagues. This study investigated a sample of female EM physicians’ current perceptions and experiences regarding breastfeeding practices and identified modifiable work-place factors affecting their decision to wean. The authors hypothesized that EM physician mothers would have excellent breastfeeding initiation rates but be largely unable to maintain breastfeeding practices upon returning to work.

**METHODS:**

A 34-item survey questionnaire evaluated demographics, perceptions, and experiences with breastfeeding with a convenience sample of EM attending and resident physicians from two Michigan academic community hospitals.

**RESULTS:**

Thirty-nine surveys were completed, representing a participant response rate of 88.6%. Breastfeeding had been initiated by all respondent mothers, all of whom returned to full-time employment after delivery. Upon return to work, 15 (75%) respondents continued to exclusively breastfeed. The goal of participants was to breastfeed for an average of 7.1 months (± 4.1 months), although the average duration children were exclusively breastfed was 5.8 months (± 4.0 months).

**CONCLUSIONS:**

Based on these results, the reasons for decreased breastfeeding after return to work in an EM residency program setting are multifactorial and include some modifiable interpersonal and institutional influences. These findings support the implementation of work-place strategies and policies to promote successful breastfeeding practices among EM resident and attending physician mothers returning to work.

## INTRODUCTION

Balancing a career and family life is a challenge many physicians face, although it can be particularly challenging for female physicians.^1,2^ Pregnancy during medical training or early years as an attending physician is common, given these times often coincide with a woman's reproductive potential. Emergency Medicine (EM) remains a male-dominated specialty (i.e., approximately 75% male) despite females accounting for half of all medical students.^3^ Attrition rates among female EM residents are also disproportionately higher than their male counterparts, with health/family being more commonly cited reasons for attrition among female EM residents.^4^

The demand for board-certified EM physicians continues to grow due to the aging population of the physician workforce and poor access to timely primary care, contributing to emergency department (ED) overutilization and overcrowding.^5^ EM has the potential to attract female physicians who desire a more sustainable work-life balance, given predictable scheduling and lack of on-call shifts.^6^ However, EM residency program officials require an improved understanding of the unique challenges female physicians may face surrounding motherhood and breastfeeding in the work-place.^7^

Postpartum employment has already been cited as a significant obstacle to breastfeeding and breastfeeding continuation rates in the general population.^8^ The challenges faced by EM physicians who desire to breastfeed can be exacerbated by long working hour requirements and lack of time to pump human milk during shifts. A 2018 study of 223 female physicians with breastfeeding experience demonstrated that 97% of respondents reported having experienced at least one barrier to their successful breastfeeding.^9^ A study of obstetric residents showed breastfeeding residents struggled with low milk supply and work demands, leading to early discontinuation of breastfeeding.^10^ A 2020 best practices article summarized these challenges identified in earlier studies.^11^

### Purpose of Study

This quantitative descriptive correlational study examined a sample of female EM physicians' perceptions and experiences with breastfeeding and attempted to identify modifiable work-place factors affecting the decision to wean. Before the study, the authors had hypothesized that EM physician mothers would have excellent breastfeeding initiation rates but would often be unable to maintain desired breastfeeding practices upon their return to work.

## METHODS

### Study Design

After IRB approval, a cross-sectional 34-question survey of respondents was developed by the authors and administered to a convenience sample of female EM physicians concerning their return to work breastfeeding experiences and perceptions. Emphasis was placed on the protection of human subjects throughout the study as survey results were anonymous, informed consent was obtained from all participants, and all data was stored on password-protected electronic storage devices throughout the study period.

### Setting

Study participants were regionally located in the metropolitan Detroit area and part of the Henry Ford Health System. Data collection occurred from May 22, 2016 to July 2, 2016 over a six week period. Surveys were administered using SurveyMonkey (SurveyMonkey, Palo Alto, CA).

### Sample

The eligible study sample consisted of a total of 44 female EM physicians employed at Henry Ford Wyandotte and Henry Ford Macomb Hospitals who had graduated from these hospitals' respective EM residency programs between 2011 and 2016.

### Data Collection & Measurement

The authors’ non-validated survey questionnaire was developed based on the instruments described in several previous publications concerning American physician breastfeeding experiences.^10,12–14^ The authors obtained information regarding respondents socio-demographic characteristics, breastfeeding experiences, and awareness of hospital breastfeeding policies. The survey questions were initially piloted by five EM physicians from Henry Ford Wyandotte Hospital to ensure clarity of questions. The final revised survey contained 34 items and took approximately 10 minutes to complete. Pilot participants were not included in the analytic sample. (see Appendix)

A recruitment letter and informed consent were emailed to 44 eligible current/past EM resident and attending physicians based on their current addresses provided by the graduate medical offices at the two health systems. Two (0.45%) graduates did not have valid contact information on file and thus were not contacted for the study.

An anonymous, self-administered online survey link was emailed to participants in May 2016. A three-week reminder email was sent to those who had not already completed a survey. The survey was accessible for a total of six weeks.

### Survey Definitions

The following two World Health Organization definitions were provided for survey respondents: *1. Breastfeeding:* "Requires that the infant receive breastmilk (including milk expressed or from a wet nurse). Allows the infant to receive anything else: any food or liquid including non-human milk and formula."^15^ 2. *Exclusive breastfeeding:* "Requires that the infant receive breast milk (including milk expressed or from a wet nurse). Allows the infant to receive oral rehydration solution, drops, syrups (vitamins, minerals, medicines). Does not allow the infant to receive anything else."^15^

### Data Analysis

Raw survey data were aggregated and analyzed. Categorical data were reported as frequencies and percentages. Continuous data were reported as means and standard deviations. A series of independent variable t-tests were used for evaluating mean differences between sample subgroups using continuous data. A series of Pearson r correlation coefficient procedures were also used to test for associations between breastfeeding duration and modifiable individual/institutional factors. SAS 9.4 software (SAS Institute Inc, Cary, NC) was used by the Henry Ford Health System biostatistics department for statistical analyses.

## RESULTS

A total of 39 (88.6%) surveys were completed. Of those respondents, 20 (51.3%) had children. For our analyses, data involving in utero fetuses and mothers actively breastfeeding were excluded. Four of the 39 participants submitted an incomplete survey. Additional participant demographic data are shown in Table 1.

**Table 1.** Participant Demographic Characteristics (N=39)

**Age (years)**	***n (%)***
27-30	13 (33.3)
31-34	18 (46.2)
35-39	5 (12.8)
≥40	3 (7.7)
**Current Marital status**	
Married	27 (69.2)
Unmarried	12 (30.8)
**Level of training**	
Resident or fellow	17 (43.6)
Attending	22 (56.4)
**Institution**	
Henry Ford Wyandotte	23 (59)
Henry Ford Macomb	16 (41)
**Other Children?**	
Yes	20 (51.3)
No	19 (48.7)

**Figure 1:**
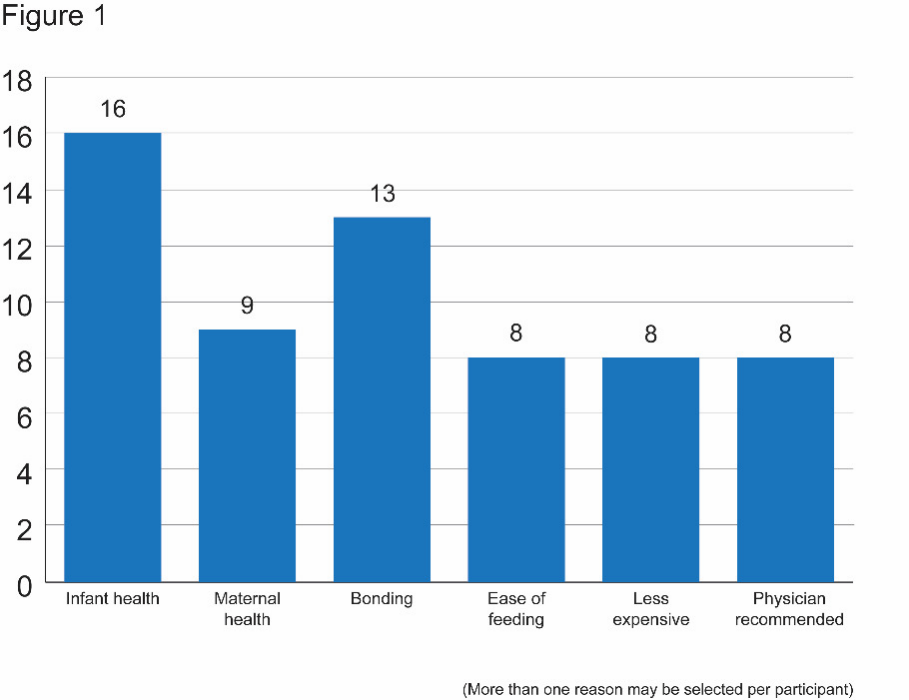
Reason for Intent to Breastfeed

All repondents reported that they had intended to breastfeed upon their child's birth. Reasons for breastfeeding included infant/maternal health, bonding, ease, and cost savings (Figure 1). Respondents breastfeeding duration goals for each child varied as follows: five (20.8%) repondents had a goal for their child to breastfeed for less than three months, six (25%) had a goal for their child to breastfeed for three-to-six months, five (20.8%) had a goal for their child to breastfeed for between seven and 12 months, and eight (33.3%) repondents had a goal for their child to breastfeed for more than 12 months. The average reported breastfeeding duration goal was 7.1 months (± 4.1 months).

**Figure 2:**
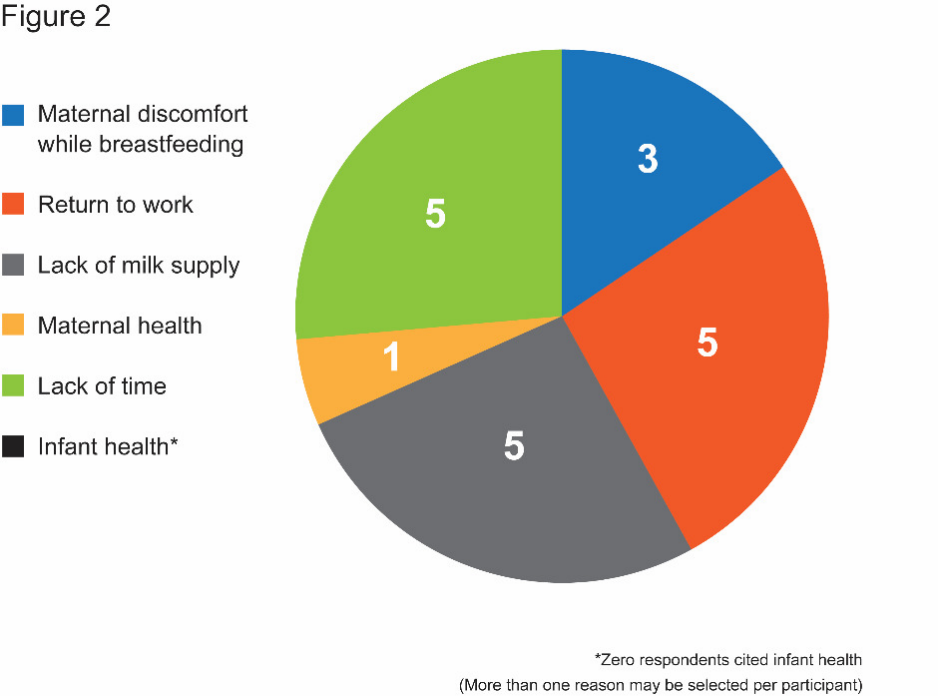
Factors influencing Decision to Wean

The average duration during which respondents’ children were breastfed was 5.8 months (± 4.0 months). A fairly strong correlation between breastfeeding duration goal and weaning age was detected (r = 0.86) (P < 0.001). Reported reasons for weaning included return to work, lack of adequate milk supply, and lack of time (Figure 2).

Continuation rates of breastfeeding at three months were 85.7% (12/14) for respondents breastfeeding their first child and 85.7% (6/7) for those breastfeeding their second child. Breastfeeding continuation rates at six months were 50% (7/14) for the first child and 57.1% (4/7) for the second child. Continuation rates at 12 months were 21.4% (3/14) for the first child and 28.6% (2/7) for the second child. A fairly high correlation was found between the weaning ages of first and second children (r = 0.72) (P = 0.071). The mean breastfeeding duration of infants during residency training was six months (± 2.6 months) compared to 7.6 months (± 3.6 months) for infants breastfed after completion of residency training. However, this difference was not statistically significant (p = 0.496).

Among respondents, all returned to full-time employment after delivery. Their average maternity leave was 9.6 weeks (± 2.3 weeks), and upon return to work, 75% (15/20) of participants continued to exclusively breastfeed. No statistically significant difference between the length of maternity leave and weaning age was detected (p = 0.621). Moreover, there was no significant correlation between the amount of support received at work and weaning age (r = - 0.035) (P = 0.909). Access to lactation-specific areas at work and weaning age showed a statistically significant difference (p = 0.050), with an older mean weaning age in children of mothers who did not feel they had have access to lactation-specific areas. Overall, 52.6% (10/19) of the participant mothers were mostly satisfied and 10.5% (2/19) of participant mothers were completely satisfied with their child's feeding regimen after returning to work. (One response missing).

Participants were also surveyed on perceptions surrounding maternity in the EM work-place. All sample respondents felt it was acceptable to have a child in residency and indicated they would try to plan to rearrange their schedule to help a pregnant colleague. However, 38.5% (15/39) of respondents felt breastfeeding colleagues placed extra demands on other EM colleagues, and 10.3% (4/39) indicated that breastfeeding colleagues had received some type of special favors. Surprisingly, 92.3% (36/39) of EM physicians were unaware of hospital policies regarding breastfeeding break policies, although many indicated a belief that such a policy was necessary to support new mothers.

## DISCUSSION

In 2020, a position paper was published outlining best practices regarding breastfeeding in EM physician mothers.^11^ Highlighted within this publication was the need for EM-specific research and outcome data to be conducted regarding this topic. Our study was apparently one of the first to explore the maternity experiences and attitudes of a sample of EM physicians.

A 2011 survey administered through Henry Ford Hospital to over 500 EM resident physicians across the country showed favorable attitudes towards pregnancy. Eighty-four percent of participants felt it was acceptable to have children during residency.^16^ Our 2016 study also demonstrated favorable attitudes towards maternity. All of our EM physician respondents thought it was acceptable to have a child during residency and reported they would expect to try to rearrange their schedules to help a pregnant colleague.

Although these findings are promising, these results demonstrate how additional breastfeeding challenges arise for many physician mothers when returning to work. A recent nationwide study of US physician mothers has reinforced the challenges that arise with breastfeeding continuation upon return to work.^17^

The *Healthy People 2020* breastfeeding initiative recognized the importance and health benefits of breastfeeding.^18^ Objectives outlined in this government initiative included goals of 60.6% and 34.1% breastfeeding continuation rates at six and 12 months, respectively.^18^ Our study results demonstrated that all respondents had initiated breastfeeding at birth, that their reported goal was to breastfeed for an average of 7.1 months (± 4.1 months). At six months, however, 52.4% (11/21) of respondents were still breastfeeding, and only 23.8% (5/21) of their children were breastfed at 12 months.

The factors influencing these lower breastfeeding continuation rates in our sample were reportedly multifactorial, with the top cited reasons including return to work-related demands, lack of time, and lack of milk supply as had been cited in earlier studies.^11,12^ In one earlier study, access to lactation facilities was also associated with breastfeeding success in working women.^19^ Only 50% (10/20) of our respondents reported knowing about available, dedicated lactation facilities at work. Mothers might be using non-designated places to pump human milk while at work.

Support from coworkers was another factor associated with increased breastfeeding duration in sample working mothers, with over 80% (17/20) of our sample women indicating that their work-place was "usually or always supportive" of breastfeeding efforts. One 2018 study demonstrated similar findings in that the most positive aspect of returning to work for physician mothers was emotional support from colleagues.^17^ However, our data also showed that 38.5% (15/39) of participants felt that breastfeeding colleagues placed extra demands on them, and 10.3% (4/39) of participants felt breastfeeding colleagues received some type of special accomodation.

A large, cross-sectional 2017 survey of physician mothers similarly revealed that 35.8% of women had experienced some type of workplace maternal discrimination. Of those who reported maternal discrimination, nearly 90% reported discrimination based on pregnancy or maternity leave, and approximately half reported discrimination based on breastfeeding.^20^

Other factors, including the length of maternity leave and work-place seniority, might influence breastfeeding duration upon return to work for physician mothers.^21^ Similar to our findings, two earlier studies found that breastfeeding rates were generally lower for mothers during residency compared to those who had already completed residency.^22,23^

This may be due to attending physicians often working fewer hours and having more control over their work environment and schedule, allowing them to incorporate breastfeeding practices into their work routine more easily.^24^

In our study, a policy supporting breastfeeding was considered important to many women, although only 7.7% (3/39) of respondents indicated awareness of a specific institutional policy. A 2016 initiative utilizing EM stakeholders from across the nation identified opportunities for policy improvement at an organizational level that addresses obstacles facing female EM physicians.^25^ Policy recommendations proposed supportive pregnancy, childbirth, and maternity leave practices including lactation facilities immediately adjacent to the ED to help ensure that women could leave the ED during a shift to complete breastpumping.^25^

Additionally, EM-specific research has recently been published regarding return-to-work policies for new resident parents. This includes no overnight shifts and a maximum of three shifts in a row for the first six weeks upon return to work.^26^ It is unclear to us whether these types of recommendations are a new standard in EM care environments with more frequently unpredictable work demands or how many lactating women may be aware of their existence.

### Limitations

The breastfeeding experiences of female physicians in ED settings are inherently multifaceted and complex. As such, there may be additional, unidentified factors that influence maternal experiences and breastfeeding practices. Our study's final convenience sample size may have also been too small to detect other meaningful sample subgroup differences. Similar to earlier studies, recall biases may have affected some of our questionnaire responses as we were relying on respondents’ memories of prior maternal experiences.^10,23^

Our results may not be externally generalizable to other ED settings since there may be significant variability in breastfeeding policies and protocols across other health systems and institutions. Workplace conditions that affect breastfeeding success for EM female physicians may also have evolved since our data collection in 2016.

## CONCLUSIONS

Our study examined the primary factors influencing maternal perceived breastfeeding success and duration upon return to work as an EM physician. In this sample, breastfeeding continuation rates fell below both the *Healthy People 2020* guidelines and initial self-reported participant breastfeeding goals. Future studies with larger EM samples are needed to investigate the multifactorial interpersonal and institutional influences contributing to breastfeeding patterns in hectic EM practice settings.

### Conflicts of Interest

The authors declare no conflicts of interest.

## APPENDIX

### SURVEY: Maternity Experience Among Emergency Medicine Physicians

**Eligibility:** All emergency medicine female physicians (attendings and residents) currently employed at Henry Ford Wyandotte and Henry Ford Macomb Hospitals as well as female graduates from the hospitals' respective emergency residency programs dating from 2011 to present.

#### DEMOGRAPHIC INFORMATION

1. With which training site are you affiliated (either past or present)?
  - Henry Ford Wyandotte Hospital
  - Henry Ford Macomb Hospital
2. Age (years)
  - 23-26
  - 27-30
  - 31-34
  - 35-39
  - 40+
3. Current level in training
  - Resident/Fellow
  - Attending
4. Marital status at time of study
  - Married
  - Single
5. Do you have any children?
  - Yes
  - No (skip to questions #24-27; 31-34)
6. Number of biological children?
  - One
  - Two
  - Three
  - Four
  - Five+
7. Please list when you gave birth:
  - Before medical school
  - During medical school
  - During residency
  - During fellowship
  - After completion of training

#### BREASTFEEDING EXPERIENCE (initiation, duration, supplementation, weaning)

1. Do you have any experience breastfeeding?
  - Yes
  - No
2. Please indicate if you planned to breastfeed your newborn upon delivery.
  - Yes
  - No (skip to question #19)
3. What were your reasons for wanting to do so? Select all that apply.
  - Infant health
  - Maternal health
  - Bonding
  - Ease of feeding
  - Less expensive
  - Physician recommendations
  - Other (please specify)
4. Did you have a duration goal in mind for breastfeeding?
  - Yes
  - No
5. If so, how long did you hope to breastfeed (indicate in months)?
6. After giving birth to your child, please indicate your child's primary source of nutrition.
  - Breastmilk only
  - Combination of breastmilk and formula
  - Formula only
7. If you chose to breastfeed exclusively, how long did you do so?
  - Less than 6 weeks
  - 6 weeks-12 weeks
  - 12 weeks-6 months
  - I am currently breastfeeding and my baby is ________ months old
8. If you have weaned your child from breastfeeding, when did you do so?
  - Less than 6 weeks
  - 6 weeks-12 weeks
  - 12 weeks-6 months
  - 6 months-12 months
  - 12 months+
9. What were your reasons for weaning completely from breastmilk? (Please select up to three responses.)
  - Mother not comfortable with breastfeeding
  - Return to work
  - Lack of adequate milk supply
  - Lack of time to pump or express breastmilk
  - Maternal health
  - Infant health
  - Infant not interested in breastfeeding
  - Other
10. If applicable, how did you deal with low milk supply? (Select all that apply)
  - Added a pumping session
  - Supplemented with formula
  - Supplemented with frozen breastmilk
  - Took herbal supplements
  - Stopped pumping
  - Not applicable
11. How long was your maternity leave? ( ____weeks)
12. When you returned to work, did you return to full-time or part-time employment?
  - Full-time employment
  - Part-time employment (<20 hours/week)
13. When you returned to work, did you continue to breastfeed your infant exclusively (pumping/milk expression)?
  - Yes
  - No
14. Did you have time for milk expression/pumping at work?
  - Never
  - Occasionally
  - Sometimes
  - Often
  - Always
  - Not applicable
15. Were lactation facilities available at your worksite?
  - Yes
  - No

#### PERCEPTIONS: Personal and Colleague

1. Do you feel it is acceptable for a female to have a child during her emergency medicine residency?
  - Yes
  - No
2. Do you feel it is acceptable for a female to have more than one child during her emergency medicine residency?
  - Yes
  - No
3. Would you rearrange your schedule to help a pregnant colleague?
  - Yes
  - No
4. Do you feel breastfeeding colleagues place extra demands on you at work?
  - Yes
  - No
5. Do you feel breastfeeding colleagues receive "special favors?"
  - Yes
  - No
6. If you had a child and breastfed when you returned to work, rate the support you received for breastfeeding efforts by fellow colleagues:
  - Always opposed my efforts
  - Usually opposed my efforts
  - Neutral
  - Usually supportive
  - Always supportive
  - Not applicable
7. If opposition was experienced, choose the following reason(s) you believed may have played a role. Select all that apply.
  - Changes in the schedule
  - Perceived special favors
  - Lack of administrative support
  - More work for others
  - Other (please specify)
  - Not applicable
8. Rate your satisfaction with your child-feeding behaviors after returning to work.
  - Completely satisfied
  - Mostly satisfied
  - Neutral
  - Dissatisfied

#### POLICY AWARENESS

1. Are you aware of any hospital policy supporting reasonable breaks for breastfeeding mothers?
  - Yes
  - No
2. Do you feel a breastfeeding policy supporting new physician mothers is important?
  - Yes
  - No
3. How many times do you feel a mother should pump during an 8-12 hour shift?
  - one
  - two
  - three
  - four
  - five
4. How many times do you feel a mother should pump on a 24-hour shift?
  - one to three
  - three to five
  - six to eight
  - nine+
